# Benefits of dietary supplements on the physical fitness of German Shepherd dogs during a drug detection training course

**DOI:** 10.1371/journal.pone.0218275

**Published:** 2019-06-14

**Authors:** Laura Menchetti, Gabriella Guelfi, Roberto Speranza, Pasquale Carotenuto, Livia Moscati, Silvana Diverio

**Affiliations:** 1 Laboratory of Ethology and Animal Welfare (LEBA), Department of Veterinary Medicine, Perugia University, Perugia, Italy; 2 GdF (Military Force of Guardia di Finanza), Dog Breeding and Training Course, Castiglione Del Lago (PG), Italy; 3 Istituto Zooprofilattico Sperimentale dell’Umbria e delle Marche, Perugia, Italy; University of Illinois, UNITED STATES

## Abstract

A high standard of physical fitness is an essential characteristic of drug detection dogs because it affects not only their ability to sustain high activity levels but also their attention and olfaction efficiency. Nutritional supplements could improve physical fitness by modulating energy metabolism, oxidative processes, and perceived fatigue. The aim of this study was to investigate the physiological and biochemical changes induced by submaximal exercise on drug detection dogs (German Shepherd breed) and to assess whether a dietary supplement improves their physical fitness. During a drug detection dog training course, seven dogs were fed with a basal diet (Control Group) for three-month period, while a further seven dogs were fed with a basal diet as well as a daily nutritional supplement containing branched-chain and limiting amino acids, carnitine, vitamins, and octacosanol (Treatment Group). At the end of this period, individual physical fitness was assessed by making each subject take a graded treadmill exercise test. A human heart rate monitor system was used to record the dog’s heart rate (HR) during the treadmill exercise and the subsequent recovery period. The parameters related to HR were analysed using nonparametric statistics. Blood samples were collected before starting the nutritional supplement treatment, before and after the treadmill exercise and following recovery. Linear mixed models were used. The dietary supplements accelerated HR recovery, as demonstrated by the lower HR after recovery (P<0.05) and Time constants of HR decay (P<0.05), and by the higher Absolute HR Recovered (P<0.05) recorded in the Treatment group compared with the Control dogs. The supplemented dogs showed the lowest concentrations of creatine kinase (CK; P<0.001), aspartate aminotransferase (AST, P<0.05) and non-esterified fatty acids (NEFA; P<0.01) suggesting a reduction in muscle damage and improvement of energy metabolism. These data suggest that this combined supplement can significantly enhance the physical fitness of drug detection dogs.

## Introduction

Since ancient times, working dogs have been considered as extremely accurate and flexible extensions of mankind’s senses and capabilities. Despite modern technological advancements, machines fail to match the competence of dogs trained for a range of tasks, i.e. for detecting explosives and narcotics or searching for missing people and avalanche victims [1–5]. For this reason, drug detection dogs are used by law-enforcement authorities all over the world [6].

However, narcotic detection is a complicated and unexplored science. The dog’s skills and disposition must match with appropriate training, with the handler’s abilities as well as with the entire dog-handler team. There are multiple physical and behavioural traits that are essential for detection dogs. Detection work requires dogs to have social-cognitive skills, innate characteristics and maintain engagement and motivation [6]. However fitness and physical training are equally essential requisites [6–8]. Detector dogs should be athletic and be trained to work long hours in physically challenging and complex search environments. They should work quickly but without missing the intended targets or exhausting themselves prematurely [6,8]. Indeed, extreme physical activity and high temperatures that result in overheating divert the dogs’ attention away from the scent detecting task (sniffing) towards maintaining their body temperature within normal limits through panting. Since dogs are unable to sniff and pant simultaneously, panting decreases sniffing rate and consequently olfaction and detection efficiency [6,8]. From a behavioural point of view, fatigue can negatively affect the strategies used by dogs in response to task demands, thus reducing the effectiveness of problem-focused coping and attention and amplifying emotion-focused coping at the same time [9].

Similarly to athletes, nutritional supplements can improve the fitness of working dogs. A recent meta-analysis by Bermingham et al. [10] showed that energy requirements of working dogs are only marginally lower than those of racing dogs. However, they did not consider specific requirements such as micronutrients and did not distinguish between the various breeds of working dogs. Some studies have evaluated the effect of supplementation on aged dogs [11,12], dogs with particular pathologies or deficiencies [13–15] or on specific aspects such as poor reproductive performance [16,17]. Vassalotti et al. [18] recently suggested that the athletic performance of search and rescue dogs may benefit from n-3 PUFA supplementation. However, previous studies have mainly been carried out on sled dogs or racing Greyhounds [19–22] while few studies have focused on the importance of quality nutrition and supplementation for working dogs [10].

Physical fitness has been defined as “the ability of the organism to maintain various internal equilibria as closely as possible to the resting state during strenuous exertion and to restore promptly after exercise any equilibriums that have been disturbed" [23,24]. This study mainly focuses on exercise-induced physiological changes as indices of physical fitness. Heart rate is a good indicator of fitness and has been used to evaluate the physical fitness of dogs in several experimental settings [1,4,7,23,25–27]. Some mathematical models of heart rate kinetics in response to exercise [28], have not only been used to evaluate animal fitness and cardiovascular health but also for assessing physical performance and the effectiveness of sports and fitness training programs. For example, the anaerobic threshold measures the ability to perform at high intensity for prolonged periods of time [29] while the Absolute HR Recovery (HRR) and Time constants of the post-exercise HR decay (τ20) are indices of heart rate recovery [4,30,31]. Many biochemical parameters are also useful for assessing the degree of physical exertion, wellbeing and recovery potential of dogs after exercise. Increases in plasma muscle enzymes such as creatine kinase (CK), aspartate aminotransferase (AST), and lactate dehydrogenase (LDH) can indicate muscle damage and fatigue [4,5,7,23,32–37]. Indeed, exercise could result in increased membrane permeability, linked to the increase in oxidant species, or membrane breakage at the level of the sarcolemma and Z-disks. [35,37]. These localized damages result in the release of muscle enzymes into the circulation that, in healthy subjects, could be transitory and reflect the physiological response to an incremental exercise test [32,36,37]. However, the degree to which serum enzymes increase is affected by several factors such as age, breed, gender, exercise type, training and also nutritional interventions [37–39]. Then, they have been used in the dog not only for the diagnosis of pathological conditions such as myopathies [38,40], but also to study physiological adaptations [5,33,36]. Moreover, changes in glucose and non-esterified fatty acids (NEFA) can indicate glycogen storage capacity in muscle and, more generally, the animal energy balance [4,5,23].

In the present study, we hypothesize that a dietary supplementation, modulating the energy metabolism, oxidative processes and perception of fatigue, could improve the physical fitness and ultimately help drug detection dogs to sustain their training course and their future work. We assume biochemical and cardiovascular changes during an incremental submaximal exercise test as indicators of dogs’ physical fitness.

Then, the aim of this study was to determine the effects of a nutritional supplement, containing branched-chain and limiting amino acids, carnitine, vitamins, and octacosanol, on the physical fitness of drug detection dogs. For this purpose, an experimental protocol was developed to evaluate the physiological and biochemical responses of these dogs to treadmill exercise.

## Materials and methods

The experimental protocol of the study was approved by the Ethical Committee of the University of Perugia protocol number: n. 2018–21 and complies with the laws established by the Italian Ministry of Health. There is a standing agreement between the Italian Military Force of GdF and the Department of Veterinary Medicine of Perugia University for the ethical testing and study of GdF working dogs.

### Animals and diet

Fourteen drug detection dogs were enrolled in this study. All of them were German Shepherd dogs, 4 females and 10 males (S1 Table) which were aged between 2–3 years and weighed between 21.7–30.5 kg. All of the dogs were born and reared in the same place, at the GdF (Guardia di Finanza), Dog Breeding and Training Centre, Castiglione del Lago, Perugia, Italy. The dogs were individually housed in indoor pens (2.9 m x 2.4 m x 2.3 m) and walked and/or trained on a daily basis for approximately 4 hours. All of the dogs were physically (i.e. found to be in good health by a veterinarian and x-rayed to confirm the absence of hip dysplasia) and behaviourally tested (i.e. absence of behavioural pathologies assessed by a veterinary behaviour consultant) in order to determine their suitability as detection dogs. All of the dogs–and their handlers–participated in the same drug detection training course. The dog’s training course, lasting six months, included sessions of basic obedience, physical exercises, socialization with people and environmental cues and drug search learning tasks carried out in several operational environments. The training course is based on the motivation of the dog to play and essentially on the principles of conditioning and learning focused on the search, detection and discrimination of drugs through the sense of smell. We would like to underline that, to protect the safety of the dogs, during the training only *pseudodrugs* are always used. These are chemical derivates of real drugs (marijuana, hashish, heroin, etc.), synthesized in a secret laboratory authorized by the Italian Ministry of Health, maintaining the same smell of the drugs but not having any effect. Under operational conditions, the fact that dogs are motivated to search for drugs to obtain their "prize" (a sleeve or a ball) to play with and not for the drug itself, significantly reduces any risk of contact contamination or inhalation of real drugs.

At the beginning of the training course the 14 drug detection dogs were randomly distributed into two groups: Treatment (n = 7) and Control (n = 7) group. During all six months of the training course, both groups were fed a nutritionally complete and balanced dry dog food (S2 Table) and had ad libitum access to water. Besides, only for the last three months of the training course, the dogs of the Treatment group also received dietary supplementation composed of commercially available branched-chain amino acids (BCAA) and other limiting amino acids (LAA), carnitine, vitamins, trace elements, octacosanol (Iken Up,Teknofarma, Torino, Italy, S3 Table). The supplementation consisted of two tablets/10 kg b.w./day the first week and one tablet/10 kg b.w./day for the following 11 weeks.

### Exercise test

**Fig 1:**
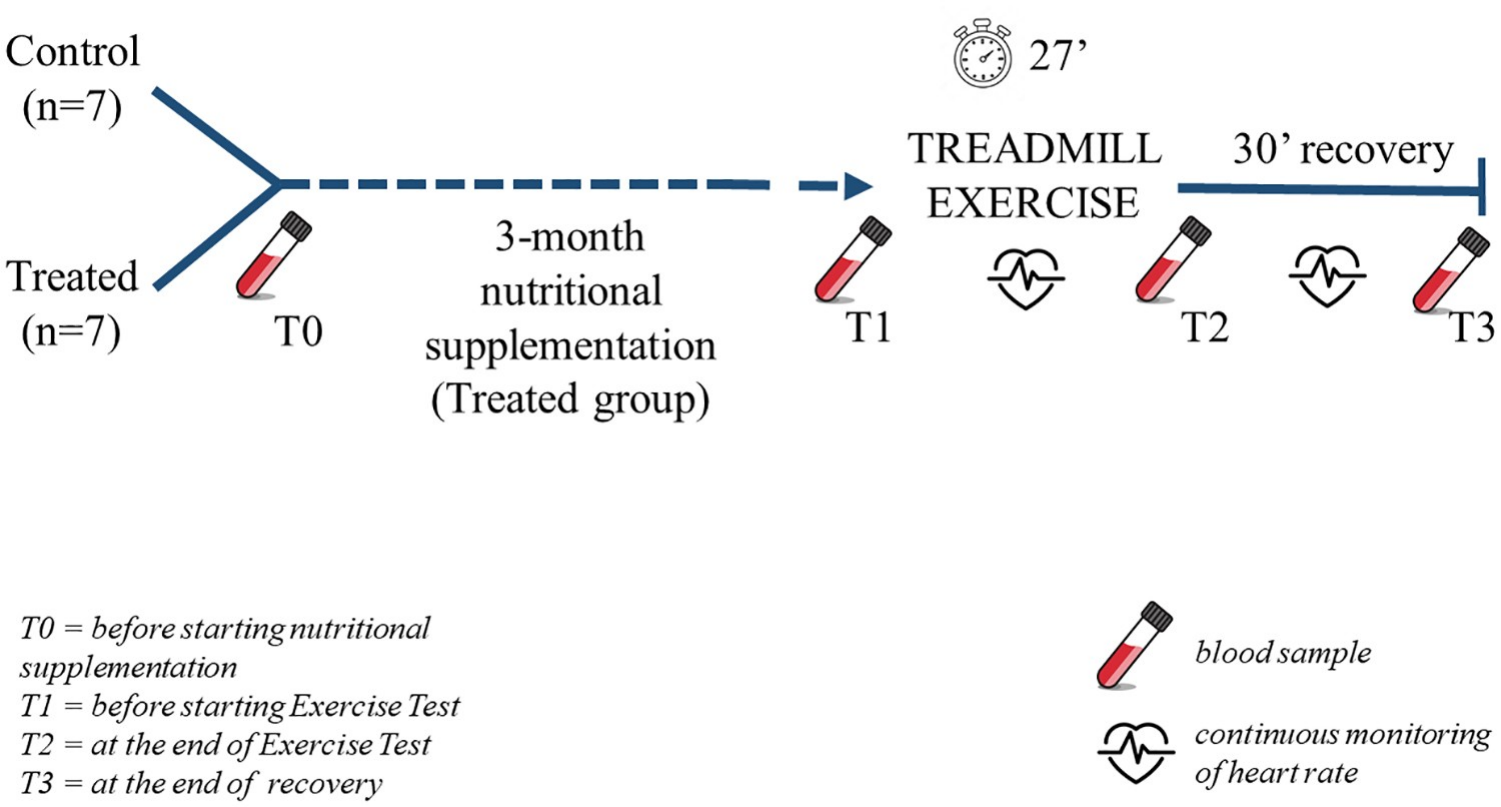
Experimental protocol. Fourteen dogs were randomly divided into two groups: a Control (n = 7) and a Treatment (n = 7) group. The dogs in the Treatment group were fed a dry diet with dietary supplementation for 3 months. At the end of this period, all of the dogs underwent a standardized submaximal treadmill Test conducted at four walking speeds and inclinations. Heart rate was monitored throughout the duration of the test (27 min) and recovery period (30 min). Blood samples were taken before starting the nutritional supplementation treatment (T0), immediately before starting the Exercise Test (T1), within the first 2 minutes after exercise test termination (T2) and at the end of the passive recovery period (T3).

At the end of the training course and dietary supplementation (Treatment group), all of the dogs were submitted to a submaximal exercise test in order to assess their physical fitness level (Fig 1). All dogs had been acclimated to treadmill experience by running 15–20 min once a week for the previous 6 months. These treadmill exercises were part of the activities included in the training course, but no specific speed protocols were foreseen. Only one dog refused to walk on the treadmill and was exempted from exercise. The Exercise Test developed for the present experimentation was performed on a motorized treadmill (professional canine treadmill, Grillo, Modena, Italy) between 9:00 and 12:00 a.m.. Both the speed and incline can be set on this treadmill. The dogs were not fed for at least 2 h before the test but were allowed to drink fresh water ad libitum. During the Exercise Test all of the handlers kept their dogs on leashes in the same way. In particular, each handler positioned in front of the treadmill keeping the dog on a leash without pulling it and providing positive vocal reinforcement to the dog.

The session (total 27 min) was conducted setting the treadmill at different walking speeds and inclinations at pre-set time intervals (S1 Fig): 5 min at a 2.5% incline at 3.8 km/h (walking, Phase 1), 5 min at a 5% incline at 3.8 km/h (walking, Phase 2), 10 min at a 2.5% incline at 7.0–8.0 km/h (trotting, Phase 3), 7 min of cool down at a 2.5% incline at 3.8 km/h (walking, Phase 4).

### Physiological parameters

Blood samples (5 ml) were taken from the saphenous vein of each dog before starting nutritional supplementation treatment (T0), immediately before the Treadmill Exercise Test (T1), within the first 2 minutes from exercise test termination (T2) and 30 min after Exercise test termination, considered as passive recovery period (T3; Fig 1). Glucose, aspartate aminotransferase (AST), alanine aminotransferase (ALT), lactate dehydrogenase (LDH), non-esterified fatty acids (NEFA), and creatine kinase (CK) were analysed using an automatic chemistry bio-analyser (Konelab 200, Italy). The analytical methods and reagents (CK, AST and LDH: Sclavo Diagnostic Dasit-Italy; NEFA: Randox, Crumlin, UK) and measure units adopted were designed for this instrument.

Rectal temperatures were assessed prior to treadmill exercise, immediately after and 30 min post-exercise using a digital thermometer (MB TERMO7126500, Reckitt Benckiser SpA, Milano, Italia).

Fifteen minutes before starting the treadmill exercise, the dogs were equipped with a Polar System (PolarM400’s built-in GPS, Polar H7 heart rate sensor with soft elastic strap; Polar Electro Oy, Kempele, Finland). Use and reliability of the Polar system for dogs was described earlier [4,27,41,42]. On the day before the Exercise test, the coat of each dog was clipped at all electrode sites, the skin was cleaned with alcohol and air-dried and Fiab electrode transmission gel (FIAB SpA–Firenze, Italy) was applied liberally to each clipped area in order to enhance conductivity. The electrode belt was strapped ventrally and the electrodes were placed on each side of the sternum. Heart rate (HR) was registered second by second and transmitted to a computer via USB cable at the end of each recording. All Polar data were provided as GPX or CSV files and transferred onto Excel spreadsheets. The Polar device was removed at the end of the 30-minute passive recovery period (T3). However, the HRs recorded during the last ten minutes of the recovery period were not considered because it was the time the dogs required for displacements. Then, we analysed 20 minutes of recovery [43].

Baseline HR was obtained from each dog the day before the treadmill exercise session (at rest), by monitoring the subjects, relaxed but awake, for 15 minutes. A five-minute interval (from min 8 to 13) was extracted and analysed [28,41].

### Data processing

Heart rate (HR) measured by the Polar device was expressed as means ± SD (bpm) and highest (HR_peak_) values.

**Fig 2:**
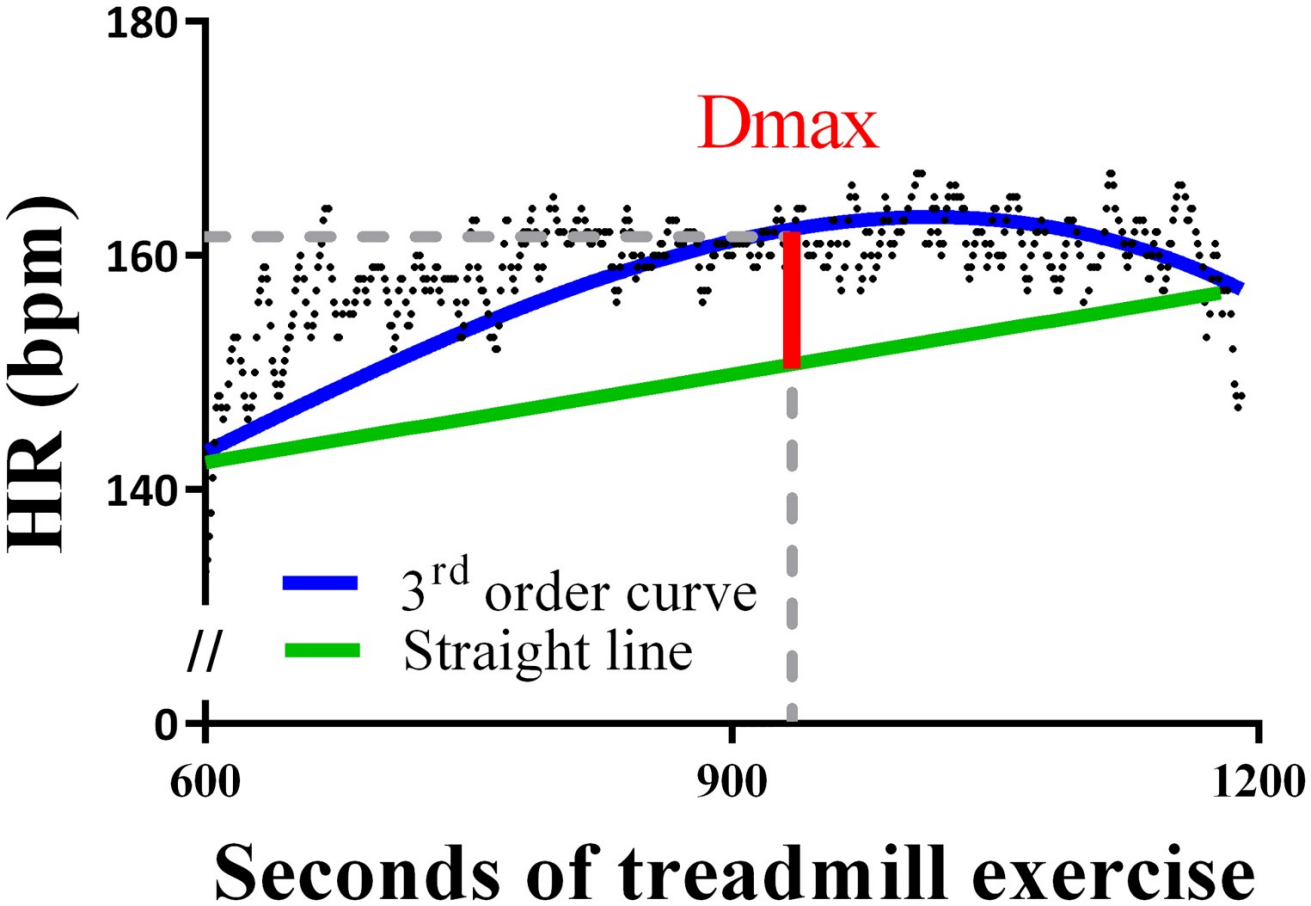
Single dog’s deflection point (HRdp; dashed line) determined via Dmax method (Dog n° 12). For each dog, a third order curve was calculated by plotting heart rate (HR) values vs time (during the incremental exercise phase); two end points of the curve were connected by a straight line and the most distant point of the curve to the line (Dmax) was considered as the HRdp.

The deflection point (HRdp) was estimated by the third-order curvilinear method (Dmax method) [44,45]. In brief, the third order curvilinear regression curve was calculated from HR values vs time (during the incremental phases of the exercise, i.e. Phases 1–3). Two end points of the curve were connected by a straight line and the most distant point of the curve to the line (Dmax) was considered as the HRdp (Fig 2) [45].

**Fig 3:**
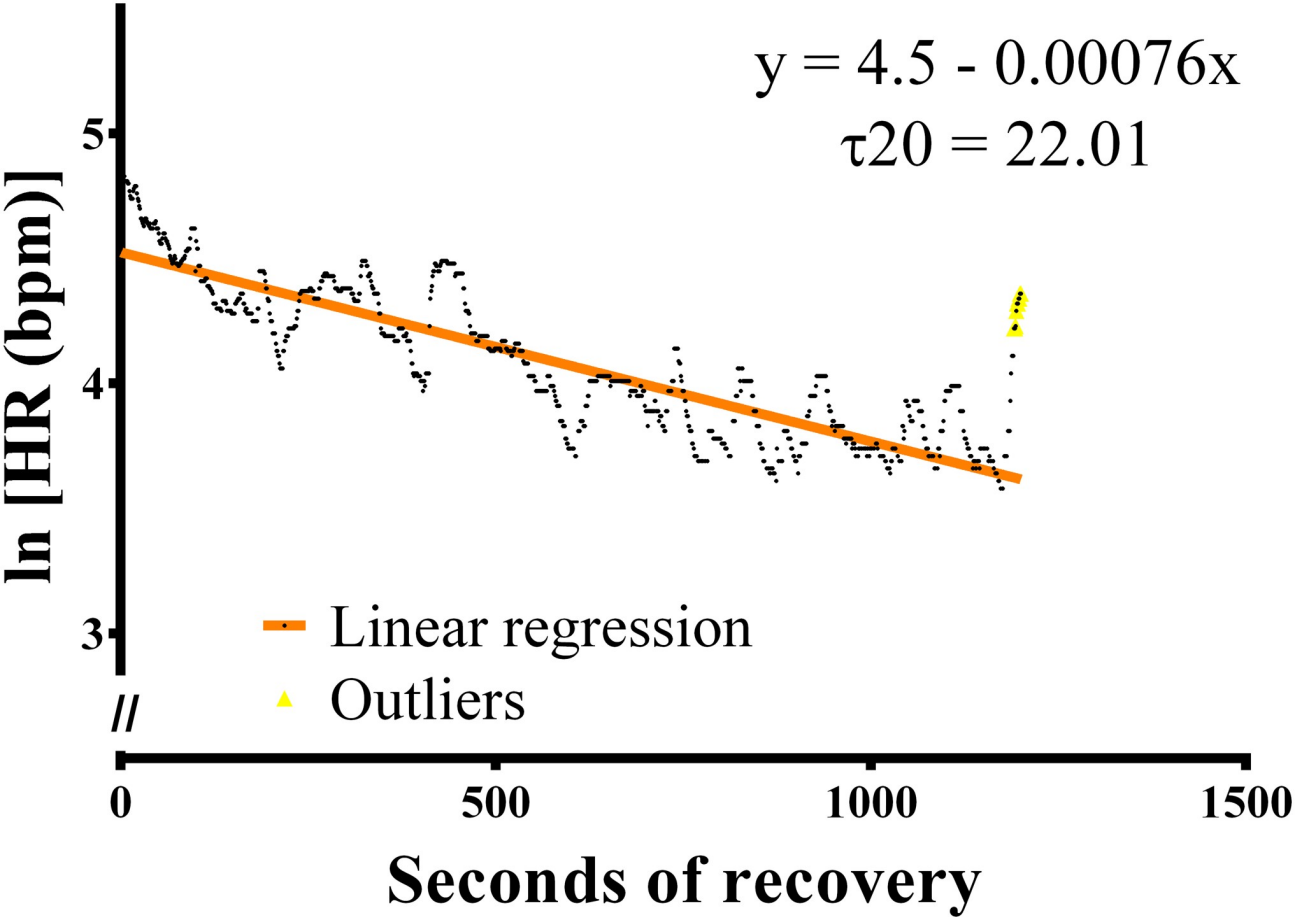
Single dog’s time constants of the post-exercise HR decay (τ20; Dog n° 7). The τ20 was determined starting from the slope of the regression line obtained by plotting the natural logarithm of the heart rate (HR) vs time (during the first 20 min of recovery).

Various parameters were used to asses post-exercise heart rate recovery (HRR): HR at 20 minutes after exercise termination, Absolute Heart rate recovered (HRR), and Time constants of the post-exercise HR decay (τ20). HRR was defined as the difference between maximal HR and HR at the end of recovery [46]. End-recovery HR was calculated as the mean value of the 20^th^ minute of recovery. The τ20 was determined as proposed by Imai et al. [47]. The natural logarithm of the HR during the first 20 min post-exercise was plotted against the time of recovery and a linear regression analysis was applied (Fig 3). The τ20 was therefore determined as the negative reciprocal of the slope of the regression line and lower values were deemed desirable [30,31,47].

### Statistical analysis

Mann-Whitney, Wilcoxon or Friedman tests were used to compare body weight, rectal temperatures and the parameters related to HR. The results were expressed as median (Mdn) and interquartile range (IQR).

Blood parameters were analysed with linear mixed model procedures. In these models, the dogs were treated as random factors while Time was treated as a repeated factor. The models evaluated the effect of Time (3 levels: T1, T2, and T3), group (2 levels: Control and Treatment groups), and interaction between time and group while the values at T0 were included as covariates. The results were expressed as estimated marginal means ± standard error (SE).

Statistical analyses were performed with SPSS Statistics version 23 (IBM, SPSS Inc., Chicago, IL, USA). Statistical significance occurred when P < 0.05.

## Results

### Body weight, Baseline HR and rectal temperature

No significant differences were observed in body weight (P = 0.209 and P = 0.259 before and after the nutritional treatment, respectively), basal heart rate (P = 0.209) and rectal temperatures (P = 0.383; S4 Table) between groups.

The rectal temperature increased after exercise (Mdn = 38.7 C°, IQR = 38.3–39.0 C° and Mdn = 39.2 C°, IQR = 38.9–39.5 C° before and after treadmill, respectively; P <0.01) and then returned to baseline values after the recovery phase (Mdn = 38.8 C°, IQR = 38.6–39.0 C°; P <0.01). However, no significant differences were observed between the two groups (S4 Table).

### HR during treadmill exercise and recovery

Regardless of group, mean HR increased from Phase 2 to Phase 3 (from walking to trotting; P = 0.01; S5 Table), while mean HR decreased from Phase 3 to Phase 4 (from trotting to walking; P = 0.003) and from Phase 4 to passive recovery (P = 0.003; S5 Table).

No significant differences were observed between study groups regarding HR means, HR_peak_ and parameters related to the HRdp obtained during the Exercise test (Table 1 and S4 Table).

**Table 1:** Heart rate (HR) mean, deflection point (HRdp), HR at 20 min after exercise termination, heart rate recovery (HRR) and time constants of the post-exercise HR decay (τ20) in control and treated groups. The values are medians and interquartile range.

Parameter	Group		P value
	Control	Treated	
**HR mean during treadmill (bpm)**	140 (110, 148)	127 (118, 152)	0.358
**HR**_**peak**_ **during treadmill (bpm)**	167 (136, 193)	161 (146, 190)	0.895
**Percentage of HR**_**peak**_ **during treadmill (%)**	80.9 (74.9, 83.9)	84.0 (78.4, 86.3)	0.181
**Time on the treadmill at HRdp**^1^ **(sec)**	947 (928, 949)	883 (852, 948)	0.177
**HRdp (% HR**_**peak**_**)** ^2^	94 (93, 95)	95 (94, 96)	0.126
**HR mean during recovery (bpm)**	96 (85, 124)	92 (63, 101)	0.234
**HR at 20 min after exercise termination (bpm)**	108 (80, 111)	84 (78, 91)	**0.012**
**HRR**^3^ **(bpm)**	48 (41, 80)	81 (70, 95)	**0.035**
**τ20**^4^ **(min)**	138 (105, 386)	57 (27, 83)	**0.008**

Bold P-values are significant at the 0.05 level.

^1^ HRdp = Heart rate deflection point

^2^ Heart rate deflection point expressed as a percentage of the maximum heart rate (HR_peak_)

^3^ HRR = Absolute Heart rate recovered

^4^ τ20 = Time constants of the post-exercise HR decay

The HRdp could not be calculated in two of the 13 dogs (15%) as their third order regression curves did not show the downward inflexion. However, these models fit well in most cases: the third order curves explained more than 50% of the variability of the data (R^2^>0.5) in 11 out of 13 dogs (S6 Table). Regardless of group, median HRdp was 95% of the maximum average heart rate (IQR = 94–96%; mean = 94%; range = 84–98%).

During the recovery phase, the Treatment dogs showed higher HRR (P = 0.035) and lower τ20 values (P = 0.008; Table 1) than those of the Control group. Twenty minutes after the end of the Exercise test (T3), HR was lower in the Treatment group than for the Control dogs (P = 0.012; Table 1).

### Blood parameters

All biochemistry and haematology parameters fell within the normal range for both groups. However, estimated marginal means recorded in the Treatment group were lower for AST (P<0.05), NEFA (P<0.01), and CK (P<0.001) than Control (Table 2). Pairwise comparisons showed differences between groups after two months of treatment (T1) and exercise (T2) for CK and after recovery (T3) for NEFA (P<0.05; Table 2). Time effects were observed for AST (P<0.001), Glucose (P<0.001), and CK (P<0.05; Table 2 and S7 Table).

**Table 2:** Physiological parameters evaluated in control and treatment groups before starting nutritional supplementation (T0), before starting the treadmill exercise (T1), within 2 minutes after completing the treadmill exercise (T2) and after 30 min of passive recovery (T3). The values (raw data) are estimated means±standard error.

Parameter	Time	Group		P value		
		Control	Treatment up	Group	Time	Group x Time
**AST (U/l)**	**T0**	24.5±3.5	24.1±6.3	**0.049**	**<0.001**	0.945
	**T1**	25.9±0.8	24.2±0.8			
	**T2**	26.4±1.1	25.3±1.1			
	**T3**	22.8±0.8	21.1±0.8			
	Mean*	**25.0**_**a**_**±0.5**	**23.5**_**b**_**±0.5**			
**ALT (U/l)**	**T0**	35.1±11.1	37.9±11.1	0.102	0.137	0.968
	**T1**	48.2±5.6	40.1±5.6			
	**T2**	49.1±6.1	40.5±6.1			
	**T3**	38.3±4.8	32.3±4.8			
	Mean*	45.2±3.2	37.6±3.2			
**Glucose (mmol/l)**	**T0**	3.9±0.4	3.6±0.4	0.630	**<0.001**	0.467
	**T1**	5.7±0.2	5.9±0.2			
	**T2**	5.6±0.2	5.6±0.2			
	**T3**	5.1±0.1	5.1±0.1			
	Mean*	5.5±0.1	5.6±0.1			
**LDH (U/l)**	**T0**	174.7±55.0	139.81±26.1	0.454	0.392	0.969
	**T1**	83.7±11.7	79.9±11.7			
	**T2**	77.2±6.5	71.4±7.1			
	**T3**	72.2±8.6	63.6±8.6			
	Mean*	77.7±5.4	71.6±5.5			
**NEFA (mmol/l)**	**T0**	0.44±0.05	0.40±0.03	**0.002**	0.327	0.062
	**T1**	0.40±0.02	0.36±0.02			
	**T2**	0.42±0.02	0.39±0.02			
	**T3**	**0.51**_**a**_**±0.05**	**0.31**_**b**_**±0.04**			
	Mean*	**0.44**_**a**_**±0.02**	**0.35**_**b**_**±0.02**			
**CK (U/l)**	**T0**	80.3±23.0	84.47±53.5	**<0.001**	**0.011**	0.744
	**T1**	**131.5**_**a**_**±13.0**	**100.4**_**b**_**±11.1**			
	**T2**	**131.8**_**a**_**±13.0**	**95.7**_**b**_**±10.8**			
	**T3**	103.9_a_±13.0	80.8_b_±6.7			
	Mean*	**122.4**_**a**_**±5.2**	**92.6**_**b**_**±5.7**			

AST = Aspartate transaminase; ALT = Alkaline Phosphatase; LDH = Lactate dehydrogenase; NEFA = Non-esterified fatty acids; CK = Creatine kinase.

Values in the same row not sharing the same subscript are significantly different (P<0.05, Sidak correction). Bold P-values are significant at the 0.05 level.

*Estimated Marginal Means for Group effect.

## Discussion

Drug detection dogs must have good olfactory detection capabilities and behavioural traits and also need to be physically fit. Indeed, excessive physical exertion may affect concentration, olfactory capability, search duration and the performance of working dogs [8].

In our study, a graded treadmill exercise protocol (Exercise test) was developed in order to assess the effects of certain dietary supplements on the physical fitness of drug detection dogs. The Treatment dogs received a dietary supplement containing branched-chain (BCAA), limiting (LAA) amino acids, carnitine, vitamins and octacosanol during the last three months of their drug detection training course. At the end of this period, a human heart rate monitor system recorded the dogs’ heart rates during a treadmill Exercise test and the subsequent recovery period. The data recorded by the device were used to calculate mean HRs and some parameters indicating cardiovascular responses to progressive incremental exercise. Moreover, recovery capacity and exercise-related degree of muscle damage and exhaustion were evaluated with conventional biomarkers. The dietary supplement proved to have some beneficial effects on the physical fitness of the drug detection dogs by reducing their HR recovery time and indicators of muscle damage after physical exercise.

The differences observed in HR means were only due to the increase in exercise intensity over time, progressively switching from walking to trotting, thus suggesting an increase in oxygen demand [4,26]. However, the incremental Exercise test enabled us to determine the HR Deflection point (HRdp) [29,48]. The HRdp is an inexpensive, non-invasive marker for implementing training programmes and assessing human endurance capacity. Indeed, it is correlated with the anaerobic threshold, the break point between the aerobic and anaerobic metabolism, which is accepted as a measure of an individual’s ability to perform at high intensity for a prolonged period of time [28,29,49,50]. The HRdp appears as a decrease in the HR-work slope during incremental exercise [29,48] and can be determined through visual inspection or mathematical models [27,28,49]. In the present study, regression techniques were used for HRdp assessment, in order to avoid subjective interpretation [29,45]. Furthermore, unlike previous studies [27,50], we have chosen to undergo animals to a submaximal exercise test. Submaximal exercise could involve less stress for the animals tested than maximal exercise and, at the same time, induce the increase in blood lactate concentration characteristic of the anaerobic threshold [51]. Indeed, the HR peaks reached by the German Shepherd dogs of the present study (136–193 bpm) were reduced compared to those obtained during maximal exercises [27,50], but similar to those of Beagles subjected to a moderate-intensity exercise (131–209 bpm) [52]. Actually, we cannot confirm the achievement of the lactate threshold in our sample because lactate concentrations during exercise were not determined. However, most of the dogs (85%) showed the downward inflexion of the time-HR curve indicating HRdp. Radin et al. [27], who carried out the first study on HRdp in dogs, reported that HRdp was on average 80% of the maximum heart rate for Border Collies. In our study, median HRdp positioned at the 95% of the peak heart rate and no significant differences were observed between groups. This value was in accordance with that recently found by Restan et al. [50] in Beagles. The concordance was not obvious as these authors submitted dogs to a maximal exercise test reaching higher maximum HR (229–291 bpm) and, further, they used visual methods to determine HRdp. In fact, differences in the breed and animal training, exercise and the method of determination may explain any discrepancies between our finding and previous studies.

Conversely, the parameters characterising the recovery phase (Absolute HR Recovered, Time constants of the HR decay and HR at the end of the recovery) showed that post-exercise heart rate recovery of the Treatment dogs was faster than for the Control dogs.

Heart rate recovery after the end of the exercise is mediated by both sympathetic withdrawal and parasympathetic reactivation, primarily by vagal reactivation, which helps the body return to normal resting state. A fast HR recovery is associated with cardiovascular fitness and endurance capacity. On the other hand, attenuated HR recovery is indicative of exhaustion due to the lack of physical fitness or training, and it is associated with increased risk of cardiovascular events and mortality [4,30,31]. At a practical level, recovery capabilities could affect the performance of working dogs. Since drug detection operations may take a long time and require much physical effort, rapid recovery may allow the dog to resume activities quickly and in optimal physiological conditions.

CK, LDH, AST, glucose and NEFA were measured to determine the degree of metabolic and muscular effort. CK and AST are important enzymes of skeletal muscle energy metabolism and indices of muscle damage following strenuous exercise, which can damage the structure of skeletal muscle cells thus increasing membrane permeability and inducing the release of these enzymes into the circulation [7,35,53]. Changes in serum levels of muscular enzymes do not necessarily imply a disease but can indicate the adaptability of muscle to physical activity as part of the physiological response. Their serum levels can be affected by several both intrinsic and extrinsic factors. In general, increases of CK reduce with the training and increase with the intensity of exercise [4,35,36]. LDH interconverts pyruvate and lactate. Elevated LDH indicates that ATP production shifts from aerobic to anaerobic processes and is a biomarker of peripheral muscle fatigue [5,32,34]. NEFA are released from the adipose tissue in response to increased energy demand in order to maintain constant blood glucose concentrations. NEFAs are generally accepted as markers of negative energy balance and are indirectly responsible for muscle fatigue as they increase progressively during exercise when muscle glycogen concentration is low [1,5,54].

In the present study, the low values of CK, AST, and NEFA suggest reductions in changes of muscle permeability and fatigue and improvements in the physical fitness of the group of supplemented dogs [5,23,55,56].

Similar changes have been observed in search and rescue dogs supplemented with n-3 PUFA, chondroitin sulphate and glucosamine [18], in hunting dogs supplemented with betaine, L-carnitine, dietary buffers, B-vitamins and yeast [55], and in racing Greyhounds supplemented with L-carnitine [22].

Regardless of the group, no significant increases in AST and CK levels were observed immediately after exercise while a decrease in these levels was evident at the end of the recovery period compared to the samples collected at the beginning of the treadmill exercise. Previous studies have reported increases in muscle enzymes in dogs after exercise but they did not investigate muscle enzymatic activity after 30 min of passive recovery [5,7,53,55]. Rovira et al. [4] and Diverio et al. [1] found that exercise induces increases in CK, AST and LDH in search and rescue dogs but these values returned to baseline after 30 min and 2 h of rest, respectively. Conversely, Cerqueira et al. [36] found the peak in CK and AST 6h after the exercise test and returning to baseline levels after 12h. In the present study, we suppose that changes in muscle metabolism did not involve significant fibrillar disruption but a long-term monitoring (up to 12 hours) would have improved the accuracy of our evaluations.

It is important to note that changes in biochemical parameters and HR may depend on many factors such as temperature, altitude, nutrition, intensity of exercise, training, age and gender [5,7,28,41,53]. However, a standardised submaximal treadmill exercise was adopted in our study and all of the dogs were born and reared under the same conditions. All of the dogs participated in the same Training course, which consisted in a 6-month period of physical and olfactory training in the detection of illegal drug substances. Moreover, the dogs were all of the same breed (German shepherd) with slight age disparities (less than a year age gap). Sample homogeneity helps to reduce potential confounding factors. We can therefore speculate that recovery capacity was enhanced by dietary supplementation.

The dietary supplement ingredients can enhance recovery capacity and fitness by exerting their influence on energy metabolism, oxidative processes, chemical damage to tissues and fatigue substances. Many studies, primarily focused on humans and rodents, have demonstrated the beneficial effects of BCAAs on endurance capacity. BCAAs reduce the perceived mental fatigue and muscle damage, supply energy to skeletal muscles, improve cognitive performance after exercise and have beneficial effects on cardiac function [56–59]. These effects appear to be mediated by changes in membrane permeability, synthesis of serotonin and cortisol and circulating ammonia [56,58]. In dogs, infusions of BCAA prevented net protein degradation during exercise [60]. Several studies carried out on human beings reported that BCAA supplementation suppresses the increase in CK (and LDH) activity induced by exercise [56,59,61].

Some dietary supplement ingredients, such as L-Carnitine and vitamins C and E reduce hypoxia and free radical damage to muscle tissue, reduce the inflammatory response after exercise-induced muscle damage, enhance fatty acid oxidation and blood flow to tissues thus mediating rapid post-exercise recovery [39,62,63]. Some studies have evaluated the effects carnitine supplementation on the physical performance of dogs and found positive benefits regarding activity intensity, muscular strength, muscle recovery and oxidative capacity [22,62,64]. Several authors have studied vitamin E supplementation in dogs, highlighting its important antioxidant properties [15,21,65–67]. Conversely, the effect of octacosanol supplementation has been investigated in rats and humans but not in dogs [68,69].

## Conclusions

The supplement containing branched-chain and limiting amino acids, carnitine, vitamins, and octacosanol proved effective in improving the physical fitness of drug detection dogs by exerting beneficial effects on HR recovery, energy metabolism and biomarkers of muscle damage. The findings have important management applications and encourage the routine use of nutritional supplements in the feeding regime of working dogs.

The present study developed an evaluation protocol, including a mathematical model of heart rate kinetics, which was applied during the training period. This model could be implemented for routinely monitoring the physical fitness of detection dogs during their working activity. Indeed, improving the fitness of drug detection dogs could optimise their performance in many operating contexts as well as their wellbeing. Moreover, our protocol could be an easy, low-cost and non-invasive tool for evaluating the performance of other working dogs, such as search and rescue dogs, which often have to work in challenging physical environments.

## Supporting information

S1 Fig Phases of exercise test on treadmill. (TIF)

S1 Table Demographic data of the dogs. (PDF)

S2 Table Nutrient composition of base diet. (PDF)

S3 Table Nutrient composition of supplemental treatment. (PDF)

S4 Table Body weight (BW), rectal temperatures, baseline HR and HR means during the different phases of the treadmill in control and treated groups. Values are medians and interquartile ranges.
(PDF)

S5 Table Heart rate means according to the phases of treadmill test and recovery. (PDF)

S6 Table Parameters and goodness of fit of the third order regression curves used to HRdp determination in each dog. (PDF)

S7 Table Estimated marginal means ± standard error (SE) and P value of time effect. (PDF)
