# Supplementary figures and images for: Benefits of dietary supplements on the physical fitness of German Shepherd dogs during a drug detection training course
Source: PLoS One. 2019 Jun 14;14(6):e0218275. doi: 10.1371/journal.pone.0218275 (PMC6570027; doi:10.1371/journal.pone.0218275)

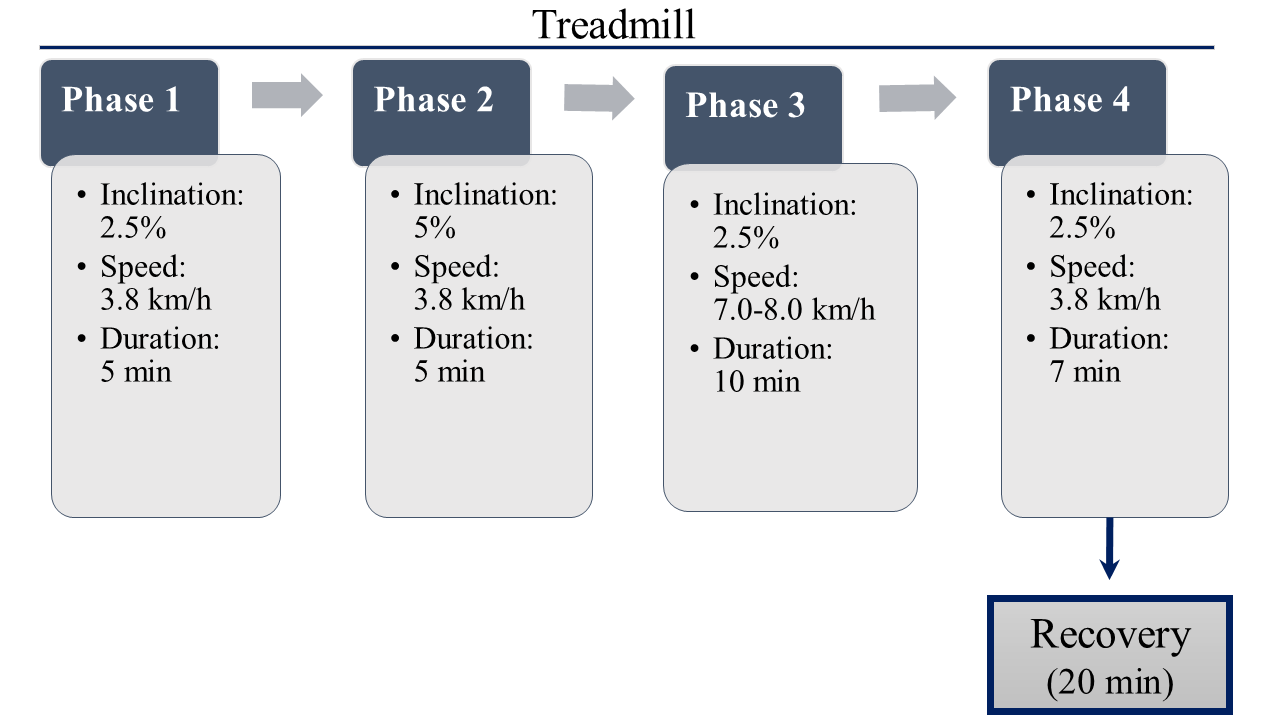

Supplement: S1 Fig — (TIF) [file pone.0218275.s001.tif]
