# Supplementary material for: Benefits of dietary supplements on the physical fitness of German Shepherd dogs during a drug detection training course
Source: PLoS One. 2019 Jun 14;14(6):e0218275. doi: 10.1371/journal.pone.0218275 (PMC6570027; doi:10.1371/journal.pone.0218275)
Supplement: S1 Table — (PDF) [file pone.0218275.s002.pdf]

S1 Table. Anagraphic data of the dogs

Group	ID	Body weight (kg)	Age (years)	Gender
Treated	1	25.4	3	♀
	2	22.7	2	♂
	3	26.1	2	♂
	4	27.3	2	♂
	5	18.2	2	♀
	6	30.5	2	♂
	7	21.7	2	♂
Control	8	25.0	2	♀
	9	28.3	2	♂
	10	26.6	3	♂
	11	26.7	2	♂
	12	30.0	2	♂
	13	27.1	3	♀
	14	26.2	2	♂
