# Supplementary material for: Benefits of dietary supplements on the physical fitness of German Shepherd dogs during a drug detection training course
Source: PLoS One. 2019 Jun 14;14(6):e0218275. doi: 10.1371/journal.pone.0218275 (PMC6570027; doi:10.1371/journal.pone.0218275)
Supplement: S2 Table — (PDF) [file pone.0218275.s003.pdf]

S2 Table. Nutrient composition of base diet

Nutrient composition	Amount
Protein (%)	26.0
Fat (%)	17.0
Ash (%)	6.5
Fibre (%)	1.2
ME* (kcal/kg)	3794
Vitamin A (UI)	15500
Vitamin D3 (UI)	1000
E1/Iron (mg/kg)	37
E2/Iodine (mg/kg)	3.7
E4/Copper (mg/kg)	11
E5/Manganese (mg/kg)	48
E6/Zinc (mg/kg)	145
E8/Selenium (mg/kg)	0.06

*Metabolizable Energy was calculated according to NRC (2006)
