# Supplementary material for: Benefits of dietary supplements on the physical fitness of German Shepherd dogs during a drug detection training course
Source: PLoS One. 2019 Jun 14;14(6):e0218275. doi: 10.1371/journal.pone.0218275 (PMC6570027; doi:10.1371/journal.pone.0218275)
Supplement: S3 Table — (PDF) [file pone.0218275.s004.pdf]

S3 Table. Nutrient composition of Supplemental treatment

Nutrient composition	Amount (mg/kg)
E6/Zinc	3300
E2/Vitamin C	16300
Vitamin E	16300
D calcium pantothenate	6500
Vitamin B1	1630
Vitamin B2	1630
Vitamin B6	820
Vitamin B12	16.3
L-Carnitine	65400
L- Lysine	41000
D,L-Methionine	41000
L-Isoleucine	39000
L-Arginine	39000
L-Valine	49000
E8/Selenium	6.5
E1/Carbonato ferroso	6845
Octacosanol	185
