# Supplementary material for: Benefits of dietary supplements on the physical fitness of German Shepherd dogs during a drug detection training course
Source: PLoS One. 2019 Jun 14;14(6):e0218275. doi: 10.1371/journal.pone.0218275 (PMC6570027; doi:10.1371/journal.pone.0218275)
Supplement: S4 Table — Values are medians and interquartile ranges. (PDF) [file pone.0218275.s005.pdf]

S4 Table. Body weight (BW), rectal temperatures, baseline HR and HR means during the different phases of the treadmill in control and treated groups. Values are medians and interquartile ranges.

**Parameter**	**Group**		**P value**
	**Control**	**Treated**	
**BW (kg) before starting the nutritional treatment (T0)**	26.7 (26.2, 28.3)	25.4 (21.7, 27.3)	0.209
**BW (kg) after the nutritional treatment (T1)**	27.0 (26.3, 28.2)	25.8 (22.0, 27.3)	0.259
**Baseline Rectal temperatures (°C)**	38.4 (38.1, 39.0)	38.7 (38.3, 39.2)	0.383
**Rectal temperatures after treadmill (°C)**	39.2 (38.9, 39.5)	39.2 (38.9, 39.5)	1.000
**Rectal temperatures after recovery (°C)**	39.0 (38.7, 39.0)	38.7 (38.5, 38.9)	0.259
**Baseline HR (bpm)**	109 (101, 118)	95 (93, 106)	0.209
**HR mean during Phase 1 (bpm)**	125 (97, 137)	122 (117, 157)	0.620
**HR mean during Phase 2 (bpm)**	127 (100, 139)	123 (121, 153)	0.620
**HR mean during Phase 3 (bpm)**	159 (114, 164)	142 (138, 158)	1.000
**HR mean during Phase 4 (bpm)**	133 (105, 143)	120 (107, 133)	0.535

Phase 1: 5 min at a 2.5% incline at 3.8 km/h.
Phase 2: 5 min at a 5% incline at 3.8 km/h.
Phase 3: 10 min at a 2.5% incline at 7.0-8.0 km/h.
Phase 4: 7 min of cool down at a 2.5% incline at 3.8 km/h.
P values from Mann-Whitney tests.
