# Supplementary material for: Benefits of dietary supplements on the physical fitness of German Shepherd dogs during a drug detection training course
Source: PLoS One. 2019 Jun 14;14(6):e0218275. doi: 10.1371/journal.pone.0218275 (PMC6570027; doi:10.1371/journal.pone.0218275)
Supplement: S5 Table — (PDF) [file pone.0218275.s006.pdf]

S5 Table. Heart rate means according to the phases of treadmill test and recovery

Parameter	Phase of treadmill and recovery					P value*
	Phase 1	Phase 2	Phase 3	Phase 4	First 20 min of recovery	
**Median (bpm)**	122_b	123_b	142_c	133_b	96_a	<0.01
**IQR (bpm)**	101-136	106-136	125-163	106-139	83-113	

IQR= interquartile range

Phase 1: 5 min at a 2.5% incline at 3.8 km/h

Phase 2: 5 min at a 5% incline at 3.8 km/h

Phase 3: 10 min at a 2.5% incline at 7.0-8.0 km/h

Phase 4: 7 min of cool down at a 2.5% incline at 3.8 km/h

* P value from Friedman tests.

Values not sharing the same subscript are significantly different (P<0.05; Wilcoxon tests).
