# Supplementary material for: Benefits of dietary supplements on the physical fitness of German Shepherd dogs during a drug detection training course
Source: PLoS One. 2019 Jun 14;14(6):e0218275. doi: 10.1371/journal.pone.0218275 (PMC6570027; doi:10.1371/journal.pone.0218275)
Supplement: S6 Table — (PDF) [file pone.0218275.s007.pdf]

S6 Table. Parameters and goodness of fit of the third order regression curves used to HRdp determination in each dog.

ID dog	Parameters				Goodness of fit	
	B0	B1	B2	B3	R^2	P value
**1***	-	-	-	-	-	-
**2**	117.1	-0.002892	8.079e-005	-7.308e-008	0.256	< 0.0001
**3**	142.3	-0.09715	0.0002575	-1.418e-007	0.770	< 0.0001
**4**	131.1	-0.1462	0.0004150	-2.587e-007	0.559	< 0.0001
**5**	114.9	-0.005188	9.832e-005	-6.553e-008	0.858	< 0.0001
**6**	169.6	-0.1613	0.0003919	-2.114e-007	0.826	< 0.0001
**7**	89.0	-0.08894	0.0003651	0.8721	0.866	< 0.0001
**8**	101.9	-0.08194	0.0002591	-1.498e-007	0.898	< 0.0001
**9**	161.6	-0.1200	0.0003047	-1.602e-007	0.908	< 0.0001
**10**	140.6	-0.09608	0.0002811	-1.572e-007	0.797	< 0.0001
**11#**	65.7	-0.03111	8.776e-005	-2.731e-008	0.619	< 0.0001
**12**	131.6	-0.09618	0.0002864	-1.582e-007	0.872	< 0.0001
**13**	143.7	-0.08847	0.0002442	-1.345e-007	0.716	< 0.0001
**14#**	102.5	0.07301	-0.0001681	1.074e-007	0.117	< 0.0001

* The dog refused to walk on the treadmill and was exempted from exercise
# Not used in the HRdp determination because there is no downward inflection
