# Supplementary material for: Benefits of dietary supplements on the physical fitness of German Shepherd dogs during a drug detection training course
Source: PLoS One. 2019 Jun 14;14(6):e0218275. doi: 10.1371/journal.pone.0218275 (PMC6570027; doi:10.1371/journal.pone.0218275)
Supplement: S7 Table — (PDF) [file pone.0218275.s008.pdf]

S7 Table. Estimated marginal means ± standard error (SE) and P value of Time effect.

Parameter	Units	Time			P value for
		T1	T2	T3	Time effect
**AST**	(U/l)	25.0_{a}±0.6	25.9_{a}±0.8	21.9_{b}±0.6	**<0.001**
**ALT**	(U/l)	44.1±3.9	44.8±4.3	35.3±3.4	0.137
**Glucose**	(mmol/l)	5.8_{a}±0.1	5.6_{a}±0.2	5.1_{b}±0.1	**<0.001**
**LDH**	(U/l)	81.8±8.2	74.3±4.7	67.9±6.0	0.392
**NEFA**	(mmol/l)	0.38±0.01	0.40±0.01	0.41±0.03	0.327
**CK**	(U/l)	116.5_{a}±7.6	113.7_{ab}±7.4	92.3_{b}±4.6	**0.011**

AST= Aspertate transaminase; ALT= Alkaline Phosphatase; LDH=Lactate dehydrogenase; NEFA= Non-esterified fatty acids; CK= Creatine kinase.
Values in the same row not sharing the same subscript are significantly different (P<0.05, Sidak correction).
Bold P-values are significant at the 0.05 level.
